# Evidence summaries (decision boxes) to prepare clinicians for shared decision-making with patients: a mixed methods implementation study

**DOI:** 10.1186/s13012-014-0144-6

**Published:** 2014-10-05

**Authors:** Anik MC Giguere, Michel Labrecque, R Brian Haynes, Roland Grad, Pierre Pluye, France Légaré, Michel Cauchon, Matthew Greenway, Pierre-Hugues Carmichael

**Affiliations:** Office of Education and Continuing Professional Development, Department of Family and Emergency Medicine, Pavillon Ferdinand-Vandry, Laval University, Local 2881-C, 1050 avenue de la Medecine, Quebec City, QC G1V 0A6 Canada; Quebec Research Centre for Excellence in Aging, Research Centre of the CHU de Quebec, St. Sacrement Hospital, 1050 Chemin Sainte-Foy, Quebec City, QC G1S 4 L8 Canada; Research Centre of the CHU de Quebec, Saint-Francois d’Assise Hospital, 10 rue de l’Espinay, D6-730, Quebec City, QC G1L 3 L5 Canada; Department of Clinical Epidemiology and Biostatistics, McMaster University, 1280 Main Street West, CRL-125, Hamilton, Ontario L8S 4 K1 Canada; Department of Medicine, DeGroote School of Medicine, McMaster University, 1280 Main Street West, CRL-125, Hamilton, Ontario L8S 4 K1 Canada; Herzl Family Practice Centre, 3755 Cote Sainte Catherine, Montreal, QC H3T 1E2 Canada; Department of Family Medicine, McGill University, 5858 Côte-des-neiges, 3rd Floor, Suite 300, Montreal, QC H3S 1Z1 Canada; Department of Family Medicine, McMaster University, 118 Lake Street, St. Catharines, Ontario L8S 4 L8 Canada

**Keywords:** Clinical practice guidelines, Knowledge translation, Decision support, Evidence-based practice, Barriers, Patient-centred care, User experience, Continuing professional development, Communication competency

## Abstract

**Background:**

Decision boxes (Dboxes) provide clinicians with research evidence about management options for medical questions that have no single best answer. Dboxes fulfil a need for rapid clinical training tools to prepare clinicians for clinician-patient communication and shared decision-making. We studied the barriers and facilitators to using the Dbox information in clinical practice.

**Methods:**

We used a mixed methods study with sequential explanatory design. We recruited family physicians, residents, and nurses from six primary health-care clinics. Participants received eight Dboxes covering various questions by email (one per week). For each Dbox, they completed a web questionnaire to rate clinical relevance and cognitive impact and to assess the determinants of their intention *to use what they learned from the Dbox to explain to their patients the advantages and disadvantages of the options*, based on the theory of planned behaviour (TPB). Following the 8-week delivery period, we conducted focus groups with clinicians and interviews with clinic administrators to explore contextual factors influencing the use of the Dbox information.

**Results:**

One hundred clinicians completed the web surveys. In 54% of the 496 questionnaires completed, they reported that their practice would be improved after having read the Dboxes, and in 40%, they stated that they would use this information for their patients. Of those who would use the information for their patients, 89% expected it would benefit their patients, especially in that it would allow the patient to make a decision more in keeping with his/her personal circumstances, values, and preferences. They intended to use the Dboxes in practice (mean 5.6 ± 1.2, scale 1–7, with 7 being “high”), and their intention was significantly related to social norm, perceived behavioural control, and attitude according to the TPB (*P* < 0.0001). In focus groups, clinicians mentioned that co-interventions such as patient decision aids and training in shared decision-making would facilitate the use of the Dbox information. Some participants would have liked a clear “bottom line” statement for each Dbox and access to printed Dboxes in consultation rooms.

**Conclusions:**

Dboxes are valued by clinicians. Tailoring of Dboxes to their needs would facilitate their implementation in practice.

**Electronic supplementary material:**

The online version of this article (doi:10.1186/s13012-014-0144-6) contains supplementary material, which is available to authorized users.

## Background

Patient-centred care requires enhancing the patient-clinician relationship through compassion, empathy, trust, spirituality, and sharing of power [1,2]. Shared decision-making (SDM) structures the sharing of power between clinicians and patients by proposing joint decisions based on an understanding of the benefits and harms of all health-care options and patients’ preferences in regard to those options [3].

Patient decision aids (PtDAs) are among the strategies most often used to effectively facilitate SDM [4,5]. These tools provide patients with information on the options and research-based outcomes relevant to their health status and help clarify values regarding the benefits and harms of each option [6]. They work as “patient-directed interventions” as they actively engage patients to enhance their knowledge and health behaviour but are also “patient-mediated interventions” as they spur patients to change the behaviours of health professionals through patient-provider interaction [7]. PtDAs have been shown to improve patients’ knowledge, reduce their decisional conflict, and lower the proportion of people passive in the decision-making process or undecided [6]. While clinicians generally agree with SDM principles [8], they may be reluctant to implement SDM because they perceive it as not applicable to some patients or clinical situations or because of time constraints [9]. In addition, the distribution of PtDAs to patients in clinical settings is arduous, and many studies that relied on clinicians to refer patients to these tools report limited utilization, often because clinicians do not view the task of referring patients to PtDAs as their role [10]. However, in most of these studies, clinicians were either unexposed to any intervention [11-16], received a financial incentive for time spent prescribing PtDAs [17] or viewing the PtDAs [18], or were exposed to a limited intervention such as brief training on how to use PtDAs [19].

Training clinicians can improve the transmission of PtDAs to patients [20,21] and increase SDM implementation in practice [20,22,23]. Participation in educational meetings can, however, be challenging for busy health-care professionals, even more so if they work in remote areas [24]. Additionally, in primary care settings, evidence on the benefits and harms of diagnostic or treatment options and clinician training in SDM are needed for multiple health problems that are too numerous to be covered in a single educational meeting.

We have thus developed a series of two-page clinical summaries known as “decision boxes” (Dboxes) that integrate the best evidence from studies and knowledge syntheses to provide information on management options for medical questions that have no single best answer [25,26]. Dboxes are most appropriate in the face of situations where trustworthy guidelines should issue weak recommendations [27]. They present information in numerical, textual, and graphical formats that follow risk communication principles [28] to allow people to make their own informed decisions. They also use colours, layout, and typographic features to enhance first impressions, clarity of content, and user experience. They facilitate critical appraisal of the evidence as they describe the included studies’ design and population, and synthesize, using the grading of recommendations, assessment, development and evaluation (GRADE) approach [29], study limitations, inconsistency of results, indirectness of the evidence, imprecision, and publication bias. Dboxes are meant to help the clinician recognize equipoise and the need to share a decision with the patient and to provide the information about the risks and benefits of all the options, so clinicians are prepared for shared decision-making [30]. Dboxes are more flexible than educational meetings, as clinicians can receive them by email or access them online, then read them at their convenience, in the setting of their choice, and use them at their own pace according to their patients’ needs. Delivery of these documents can be spaced in time, which has been shown to improve medical knowledge acquisition and retention [31-33].

In an earlier study, we interviewed patients and clinicians after they had read two Dboxes and learned how they valued this source of information for clinicians [25]. From the results, we adapted the Dboxes to improve users’ first impressions as well as their understanding of and trust in the information they provide. However, this first study did not allow testing the usefulness of the Dbox information in clinical settings, so we sought insight into the barriers and facilitators influencing clinicians’ transmission of the Dbox information to their patients during the primary care consultation. In line with Moore et al. [34], the present study aimed to document one level of outcomes of continuing education, specifically the use of educational information from Dboxes in practice. Our objectives were to measure the value of and intention to use Dboxes in practice and to describe barriers and facilitators of their use. The Dboxes will subsequently be tailored to the identified barriers in order to optimize their implementation.

## Methods

### Study design

As described elsewhere [35], this project was based on the theory of mechanisms of planned change as described in the Ottawa Model of Research Use (OMRU) [36,37]. Quantitative and qualitative sequential phases of data collection and analysis framed this mixed methods study (sequential explanatory design) [38].

### Participants and recruitment strategy

Using the professional networks of the research team members, we recruited six primary care clinics: two French-speaking and four English-speaking. Among these six clinics, two declined to participate in the second phase consisting of focus groups, so they participated only in the quantitative phase. We invited all the family physicians, nurses, and residents from these six clinics to participate. For phase two, we selected a purposeful sample of eight clinicians per clinic among extreme cases on the web questionnaires, i.e. clinicians who consistently attributed higher or lower scores to the Dboxes they rated. Thus, only the participants who completed at least one of the eight web questionnaires during phase one were eligible to participate in phase two. We also recruited the medical director of each clinic.

### Intervention

The intervention consisted of eight evidence-based Dboxes (Additional file 1) on common primary care interventions (Table 1) written in both French and English. It also included a website presenting the Dboxes with a brief tutorial, as well as educational material on patient counselling and on the GRADE study quality assessment that is integrated into the Dbox approach. The participating clinicians were e-mailed one Dbox weekly for a total of 8 weeks.

**Table 1:** **Health topic covered by each Dbox**

**Order of delivery**	**Health topic covered by Dboxes (abbreviation)**
1	Cholinesterase inhibitors to reduce the symptoms of Alzheimer’s disease (ChEIs)
2	Acetylsalicylic acid for primary prevention of cardiovascular disease (ASA)
3	Faecal occult blood test to screen for colorectal cancer (FOBT)
4	Serum integrated test to screen women for fetal trisomy 21 (Prenatal)
5	Statins for primary prevention of cardiovascular disease (Statins)
6	BRCA1/2 gene mutation test to evaluate the risks of breast and ovarian cancer (BRCA)
7	Bisphosphonates to prevent osteoporotic fractures in postmenopausal women (Osteo)
8	Prostate-specific antigen test to screen men for prostate cancer (PSA)

Health topic covered by each Dbox, their abbreviated title, and their order of delivery.

### Data collection and procedures

#### Phase one: quantitative study

At study entry, all participating clinicians consented and completed a questionnaire assessing their demographic and professional characteristics (age, gender, number of years of clinical practice). They also rated their interest for each of the eight Dbox topics using a visual analog scale ranging from “no interest” to “high interest”.

When receiving a Dbox, the participants completed a three-part web-based questionnaire (available in [35]). In part I, a 5-point rating scale assessed overall satisfaction. In part II, the validated Information Assessment Method (IAM) questionnaire spurred reflection on the cognitive impact of this information, its relevance for at least one patient in the practice, its use for this patient, and if used, any expected health benefits [39]. The questionnaire also contained free text fields that provided an optional opportunity for readers to comment. Part III consisted of a 12-item questionnaire based on the theory of planned behaviour (TPB) [40], with each item scored on a −3 to +3 rating scale, to study the factors influencing their intention *to use what they learned from the Dbox to explain the advantages and disadvantages of the options to their next patient to whom this intervention might apply*. Based on the TPB, the three determinants of a clinician’s intention to perform a given behaviour are attitude towards performing this behaviour, subjective norm, and perceived behavioural control. Each determinant is one construct of the TPB questionnaire. Internal consistency (Cronbach’s alpha) for each construct was very good (0.89–0.94).

#### Phase 2: qualitative study

After the 8-week intervention, we conducted a 60-min semi-structured focus group in each clinic with a mixed group comprising family physicians, nurses, and a resident to describe how they used what they learned in the Dbox in their practice and the influence of contextual factors and team dynamics on this use (Additional file 2). A 30-min individual interview with the medical director of each clinic also provided an administrator’s perspective of the contextual factors influencing use of the Dboxes and the potential resources needed to facilitate using them. The same person (AMCG) moderated all the interviews. One observer took notes on the conduct and content of the discussions. Each discussion was audiotaped and transcribed.

### Analysis

#### Phase one: quantitative

We performed descriptive statistical analyses of the answers to the web questionnaires.

A generalized linear mixed model was used to evaluate whether clinicians’ perceptions of the Dboxes (5-point scale and IAM) were influenced by the Dbox topic, the clinicians’ profession (family physician, resident, or nurse), the clinical site, and participants’ gender. Dbox topics, clinicians’ profession, and gender were introduced as fixed effects, and the clinical site as a random effect.

For the TPB questions, we replaced missing data with the means of the values from the questions from the same construct. Repeated measure regression models were used to evaluate relationships between clinicians’ intention to use what they learned from the Dboxes and the potential determinants of their intention (attitude, subjective norm, perceived behavioural control), their profession (nurse, resident, physician), their sociodemographic characteristics (age, gender, number of years in practice), the study site, and the clinical topic of the Dbox. We also used a mixed model to test whether the determinants of the intention varied depending on the Dbox topic. All analyses were performed using SAS (Version 9.3, copyright 2002–2010 SAS Institute Inc.).

#### Phase two: qualitative

One researcher (AG) and three research assistants who had not been involved in the development of the Dboxes and the data collection performed a thematic qualitative data analysis of the focus group discussions and of the open-ended questions from the web questionnaire, following a hybrid deductive/inductive approach [41]. The deductive analysis searched for OMRU attributes [36,37] and for attributes of the steps involved in the users’ experience of an evidence-based shared decision-making support tool over time [25], or of the IP-SDM model [42]. The inductive analysis incorporated new themes mentioned by participants.

First, to assess whether the chosen models and frameworks applied and to explore possible sub-themes, the researcher and two research assistants went separately through the same portions of two of the focus group transcripts. The three coders then compared their results and came to a consensus on a number of themes. They noted these themes in a manual of codes, and labelled and defined them. The transcripts were entered as project documents into specialized software (NVivo 10, QSR International, Cambridge, MA, USA), and the codes developed for the manual were entered as nodes.

One of the research assistants then applied these preliminary codes to half the transcripts to identify meaningful units of text. To validate coding, this research assistant submitted 67 excerpts to a second assistant, who then linked each excerpt to one of the previously defined codes. For 44 of these excerpts (66%), the two assistants chose the same code. Among the 23 excerpts linked to different codes, 12 were discussed briefly and the codes were merged. For the 11 remaining codes, longer discussions led to the creation of new codes, improvement of code definitions, or merging of codes. The first assistant completed the coding of the remaining transcripts using this revised manual.

In the final step, a third research assistant reviewed the excerpts extracted for each code to ensure completeness and appropriateness of the code manual and consistency of approach. Coding was modified, as necessary, and modifications were verified by the first author (AMCG). This third assistant produced the qualitative analyses report that was incorporated to the present article without any modification.

#### Mixing quantitative and qualitative results

We interpreted quantitative and qualitative study findings together to suggest improvements to the Dbox approach in order to facilitate their implementation in clinical practice.

This project was approved by the research ethics committees of the Research Centre at Centre Hospitalier Universitaire de Quebec, Jewish General Hospital in Montreal, and McMaster University.

## Results

### Participants’ characteristics

Overall, 101 clinicians from six clinics located in four cities participated in the study, out of 187 that were invited, for an overall recruitment rate of 54%. Table 2 presents the characteristics of the participating clinics and clinicians.

**Table 2:** **Characteristics of the participating clinics and clinicians**

	**Teaching clinics?**	**Metropolitan area population**	**Number of clinicians**		
			**Physicians**	**Residents**	**Nurses**
Clinic (only C1 to C4 participated in the focus groups)					
Clinic 1 (C1)	Yes	719,200	19	21	3
Clinic 2 (C2)	Yes	719,200	23	24	3
Clinic 3 (C3)	No	390,300	6	0	0
Clinic 4 (C4)	Yes	3,635,000	20	40	9
Clinic 5 (C5)	No	390,300	10	0	0
Clinic 6 (C6)	Yes	692,900	23	18	6
Participating clinicians					
Web questionnaire					
*N* (total = 101)			60	30	11
Mean age in years (SD)			43 (10)	30 (6)	39 (9)
*N* women (% women)			38 (63%)	20 (67%)	10 (91%)
*N* English-speaking (%)			31 (52%)	9 (30%)	6 (55%)
Mean years of practice (SD)			16 (10)	4 (6)	17 (10)
Interviews and focus groups					
*N* (total = 27)			18 (3 clinic administrators)	3	6
Mean age in years (SD)			42 (10)	29 (6)	37 (5)
*N* women (% women)			10 (52%)	2 (67%)	6 (100%)
*N* English-speaking (%)			9 (47%)	0 (0%)	3 (50%)
Mean years of practice (SD)			16 (11)	5 (6)	13 (7)

### Quantitative results

#### Questionnaire completion rate

In total, 808 questionnaires were sent to clinicians (8 per clinician, 101 clinicians), and of these, 61% were completed (496 responses out of 808 questionnaires). Overall, 67% of the clinicians completed at least 4 of the 8 questionnaires.

#### Satisfaction with the decision box

Clinicians reported a level of satisfaction with the Dboxes of 4 or 5 on a 5-point smiley-face rating scale ranging from 1 (sad face) to 5 (smiling face) in 81% of questionnaires completed (373/463).

#### The value of decision boxes for practice

Based on IAM ratings, reading Dboxes was felt to improve clinical practice (54% of completed questionnaires), specifically in the areas of counselling and disease prevention or health education (Table 3). Clinicians reported learning something new from Dboxes in 52% of questionnaires and that the information was totally (76% of questionnaires) or partially (20%) relevant for at least one of their patients.

**Table 3:** **Clinicians’ report of the value of decision boxes for practice: ratings based on the Information Assessment Method (IAM)**

**IAM items**	**Ratings (%)**
Cognitive impact of the information	
Their practice will be changed and improved	54% (268/496)
Counselling approach	76% (203/268)
Disease prevention or health education	51% (137/268)
Therapeutic approach	33% (87/268)
Diagnostic approach	16% (43/268)
They learned something new	52% (258/496)
They are motivated to learn more	32% (157/496)
They were reminded of something they already knew	23% (114/496)
They are reassured	18% (88/496)
This information confirmed current practice	13% (63/496)
There is a problem with the presentation of this information	15% (76/496)
Poorly written	25% (19/76)
Too technical	25% (19/76)
Not enough information	18% (14/76)
Too much information	17% (13/76)
They are dissatisfied	6% (30/496)
They disagree with the content of this information	2% (10/496)
This information is potentially harmful	1% (5/496)
Relevance	
The information is totally or partially relevant for at least one of their patients	96% (472/489; 7 missing)
Information use (for participants who reported the information to be totally or partially relevant)	
They will use this information for a specific patient	40% (190/472)
To discuss with patient or with other health professionals	65% (123/190)
To change the way they manage a patient	24% (45/190)
To justify a choice	24% (45/190)
To be more certain about the management of a patient	19% (37/190)
To better understand a particular issue related to a patient	12% (23/190)
To persuade a patient or other health professionals to make a change	8% (15/190)
To decide how to manage a patient	8% (16/190)
Expected benefits of the information (for participants who reported that they will use this information for a specific patient)	
They expect patient health benefits as a result of applying this information	89% (166/186; 4 missing)
Allows the patient to make a decision that is more in line with his/her personal circumstances, values, and preferences	72% (120/166)
Helps to avoid unnecessary or inappropriate treatment, diagnostic procedures, preventive interventions, or a referral for this patient	38% (63/166)
Helps reduce the patient’s uncertainty about the best decision to make	28% (47/166)

IAM is a checklist, and users are instructed to check all the items that apply (answers are not mutually exclusive).

Of the questionnaires where clinicians reported Dbox information to be totally or partially relevant, 40% reported that they would actually use this information for a patient, and the most frequently reported planned use was in a discussion with a patient or with other health professionals about a patient.

The Dbox clinical topics themselves influenced whether clinicians thought their practice would be improved after receiving the Dboxes, with maximal values obtained for the cholinesterase inhibitors to reduce the symptoms of Alzheimer’s disease (ChEIs) Dbox and minimal values for the Osteo Dbox (Table 4). Clinical topics also influenced perceptions of the relevance of the information provided in the Dbox (with maximal values obtained by the Prenatal Dbox and minimal values by the BRCA1/2 gene mutation test to evaluate the risks of breast and ovarian cancer (BRCA) Dbox) and perceptions of problems with the information, with more problems perceived with regard to the acetylsalicylic acid for primary prevention of cardiovascular disease (ASA) Dbox and fewer problems with the Prenatal Dbox. The clinicians’ profession influenced perceptions of problems with the information: nurses reported higher levels of satisfaction, while physicians reported more problems with the information. We observed no influence of either the clinical site or gender on clinicians’ perceptions of the Dboxes.

**Table 4:** **Results of the generalized linear mixed modelling to evaluate the influence of independent factors (clinicians’ profession, Dbox clinical topic, clinical site, gender) on the explanatory variables of clinicians’ perception of the value of the Dbox for practice (satisfaction with regard to Dbox, IAM value of the information)**

	***P*** **value**			
	**Profession effect**	**Dbox clinical topic effect**	**Clinical site effect**	**Gender**
	**Fixed effect**	**Fixed effect**	**Random effect**	
Smiley-face rating scale				
Satisfaction with regard to the Dbox	n.s. (*P* = 0.054)	n.s. (*P* = 0.15)	n.s. (*P* = 0.15)	n.s. (*P* = 0.4)
IAM				
Their practice will be changed and improved	ns. (*P* = 0.24)	*P* < 0.01	n.s. (*P* = 1.0)	n.s. (*P* = 0.86)
Are dissatisfied	n.s. (*P* = 0.052)	n.s. (*P* = 0.95)	n.s. (*P* = 1.0)	n.s. (*P* = 0.41)
Problem with the presentation of the information	*P* < 0.05	*P* = 0.01	n.s. (*P* = 1.0)	n.s. (*P* = 0.85)
Relevance	n.s. (*P* = 0.22)	*P* < 0.0001	n.s. (*P* = 1.0)	n.s. (*P* = 0.98)
Information use	n.s. (*P* = 0.29)	n.s. (*P* = 0.32)	n.s. (*P* = 1.0)	n.s. (*P* = 0.20)
Expected benefits	n.s. (*P* = 0.26)	n.s. (*P* = 0.75)	n.s. (*P* = 1.0)	n.s. (*P* = 0.05)

*n.s.* not significant.

#### Intention to use the decision box information in practice

The clinicians’ intention *to use what they learned from the Dbox to explain the advantages and disadvantages of the options to their next patient to whom this intervention might apply* averaged 5.6 on a scale of 1 (strongly disagree) to 7 (strongly agree), indicating that they had the intention to use what they learned. The clinicians’ intention was significantly related to social norm (*P* < 0.0001), perceived behavioural control (*P* < 0.0001), and attitude (*P* < 0.0001). For an increase of 1 point in each of “social norm,” “perceived behavioural control,” or “attitude,” we obtained an increase in intention of 0.2, 0.5, and 0.4 points, respectively, meaning that among the three determinants, perceived behavioural control has the most influence on this intention.

**Figure 1:**
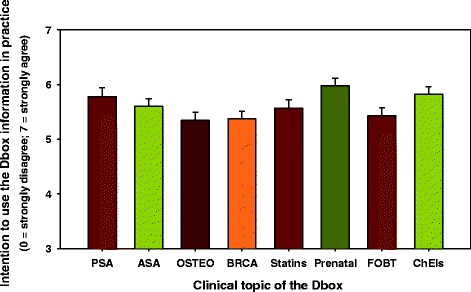
**Mean intention to use the decision box information in practice (+standard error), as measured using the theory of planned behaviour [** 40 **]** **for each of the eight Dboxes.** See Table 5 for statistical significant differences across Dboxes. For list of abbreviations, please see Table 1.

The clinicians’ intention to use the Dbox information was not influenced by their profession (*P* = 0.3), age (*P* = 0.4), gender (*P* = 0.2), the number of years in practice (*P* = 0.6), or the clinic (*P* = 0.8). It was, however, influenced by the topic of the Dbox (*P* = 0.002; Figure 1): clinicians had the highest intention to use the Dbox on prenatal screening for trisomy 21, closely followed by prostate-specific antigen (PSA) testing for prostate cancer and cholinesterase inhibitors for Alzheimer’s disease (Table 5).

**Table 5:** ***P*** **values of the significance of the differences in the intention to use the decision box information in practice among Dbox topics (n.s.** = ***P*** **> 0.05)**

	**PSA**	**ASA**	**OSTEO**	**BRCA**	**Statins**	**Prenatal**	**FOBT**
PSA							
ASA	n.s.						
OSTEO	0.02	n.s.					
BRCA	0.03	n.s.	n.s.				
Statins	n.s.	n.s.	n.s.	n.s.			
Prenatal	n.s.	0.03	0.0005	0.0006	0.02		
FOBT	n.s.	n.s.	n.s.	n.s.	n.s.	0.002	
ChEIs	n.s.	n.s.	0.007	n.s.	n.s.	n.s.	0.02

For list of abbreviations, please see Table 1.

### Qualitative findings

The citations mentioned in this section (c1 to c52) are reported in Additional file 3.

1. *Learning with the Dbox*
  Participating clinicians explained that Dboxes would support residents’ training, either during case reviews when a patient disagrees with recommended treatment (c1), when counselling a patient for the first time on a given topic, or during residents’ critical appraisal of medical literature training activities (c2). Some participants also mentioned how Dboxes represented brief continuing professional development (CPD) activities and that delivering them in the context of a CPD program might improve their uptake (c3). Clinicians also appreciated the figures and statistics presented in the Dboxes (c4) and many felt they complemented the information they already provided (c5). Clinicians reported that they would reconsider some medical interventions after reading the Dbox (c6), as they realized they were not as effective as they thought (c7). Some clinicians said that the evidence presented gave them a shock (c8), being depressed after reading the Dboxes, or stopping looking at them altogether (c9). A few participants mentioned that looking exclusively at scientific evidence may give a false impression that clinical decisions are inappropriate, when in fact there are many more things to consider than numbers. For instance, some clinicians mentioned that medications such as cholinesterase inhibitors might be useful not only for treatment but also to bring some hope to patients when there is no alternative treatment (c10). Others mentioned that clinical practice requires not only figures but also clinical judgement to take into account age, autonomy of the patient, and current medication, among other factors (c11).
2. *Counselling patients with the Dbox*
  Analyses of the discussions reveal that Dboxes facilitate the patient-provider discussion by empowering clinicians (i) to provide the relevant scientific information to their patients (c12), (ii) to answer patients’ questions (c13), (iii) to consider the patients’ point of view (c14), and (iv) to come up with a plan with the patient (c15).
  Clinicians mentioned that the quantitative information helped patients improve their understanding of the stakes and balance the pros and cons, thereby helping them make a better decision (c16).
  Clinicians appreciated the fact that Dboxes provide patients with more precise information on multiple reasonable choices (c17). They also mentioned how presenting scientific data helps lower people’s expectations regarding the effectiveness of an intervention, thus facilitating follow-up when a treatment fails, because it clearly lays out the lack of available evidence (c18).
  According to clinicians, sharing the figures presented in the Dboxes also allows them to go further in counselling by allowing patients to understand more difficult concepts, resulting in better patient compliance (c19). On the other hand, a few participants feared that presenting the uncertainty associated with an intervention might compromise patient compliance (c20) and increase decisional conflict (c21). Clinicians also mentioned that some patients might use other forms of logic and would not be receptive to information about risks (c22).
3. *Critical barriers to implementation: optimizing the intervention*
  3.1. *Adding a patient decision aid*
    We designed the Dboxes to be read before the consultation to facilitate the integration of the required information before the actual patient-clinician encounter. Some clinicians reported this type of use (c23). However, several clinicians considered giving the Dboxes to patients and not merely using them as training material for themselves (c24). However, the Dboxes were designed not for patients but rather as professional training tools, and accordingly most clinicians felt that the information was generally too complex to share with patients in that form (c25).
    Some clinicians mentioned that it was paradoxical that Dboxes aim to support SDM when patients could not actually use them (c26), and in all the discussions, considerable emphasis was placed on developing a simplified tool for patients (c27). To adapt the Dbox to patient needs, they suggested removing the section about confidence in the results (c28) and adding visual representations of the risks to facilitate patient understanding (c29). They also proposed developing two versions tailored to lower and higher levels of patient literacy and numeracy (c30).
    Clinicians generally felt that patients should have access to the patient decision aid before going through SDM with them to reduce consultation time and give some patients time to think about the options (c31). To implement this, there were suggestions that the information be delivered to patients in the waiting room, either in print or video format. There were also suggestions to go through the information in a pre-visit (c32).
  3.2. *Improving clarity of the information for some Dboxes*
    While many clinicians mentioned that they appreciated the Dboxes as learning and counselling tools, some specific Dboxes were criticized because they presented too much information or were difficult to understand (c33). Dboxes on bisphosphonates, ChEIs, and prenatal testing were generally perceived as easier to understand, whereas clinicians raised understanding issues for the Dboxes on BRCA, faecal occult blood test to screen for colorectal cancer (FOBT), and ASA. Additionally, a few participants mentioned that the Dboxes generally lacked interpretations or recommendations for them to consider and that they would have appreciated a more practical document that could be read and understood in less time (c34).
4. *External factors influencing Dbox use*
  4.1 *Patient preferences*
    Clinicians mentioned two types of patients for whom they would be reluctant to use the Dbox information: those who prefer that clinicians make the decision (c35) and those who have already made up their mind (c36). Clinicians also felt that patient preferences with regard to their involvement in decision making are highly variable and can depend on patients’ understanding of the information, on their age (with older people less inclined to be involved), on their literacy level and socio-demographic characteristics, on how informed they are, and on social pressure to have a certain procedure performed (c37).
  4.2 *Accessing the Dboxes*
    To facilitate clinicians’ access to the Dboxes and to address their diverse preferences, participants suggested offering various formats, including applications for smart phones and paper-based formats (c38).
  4.3 *Time*
    Clinicians mentioned that they would need some time to familiarize themselves with the Dboxes before using them in practice (c39).
  4.4 *Opinion leader*
    One of the clinic directors mentioned that having an opinion leader endorse the Dbox would be an important determinant of its successful implementation (c40).
  4.5 *Journal club*
    Having a group meeting to discuss Dboxes (e.g. a journal club) and using them for practice audit activities were perceived as helpful in facilitating the use of Dbox information in practice (c41).
  4.6 *Clinical context*
    Clinicians perceived the Dboxes to be more relevant in contexts where there is uncertainty with regard to clinical decisions (c42, c43). Since the Dboxes present mostly numbers and statistics, they were perceived as less useful in clinical contexts where there are important ethical issues at stake (c44). The sharing of the Dbox information was also perceived as more difficult with patients presenting multiple health problems, such as is often the case with older patients or with those who have not consulted for some time (c45). Participants perceived the Dboxes as less relevant when they dealt with a topic with which they were already comfortable (c47), when they were already using another information tool (c48), or where the presented options were not currently recommended by clinic staff (e.g. FOBT when everyone is recommending colonoscopy). Prior training in SDM was mentioned as an important facilitator to the use of Dboxes.
  4.7 *Organizational context (setting)*
    Some participants perceived private practices as a setting that could facilitate sharing the Dbox information with patients, as one might have more latitude, for example, to organize pre-visits. Some mentioned that working in a hospital environment was less favorable, because of a lack of support from other professionals (c49). On the other hand, some participants (themselves working in the public sector) mentioned how physicians in the private sector, paid through fee-for-service, would never have time to review a document during consultations but would rather use the figures. Clinicians from teaching clinics mentioned many facilitators for the use of Dboxes in their particular setting, such as being able to give longer consultations because they are salaried, being more aware of new trends in professional practice as they are frequently involved in research projects (c50), and needing to stay up to date because of their teaching responsibilities.
    The clinicians discussed how following-up on all the decisions made from one visit to the next would be costly to incorporate into their clinic’s processes. They also mentioned that organizing pre-clinic visits to provide counselling would require far more human resources than those available (c51). An upgrade of the computers in each consultation room would also facilitate the use of Dboxes, but the clinics would need to find additional funding for this.
  4.8 *Interprofessional approach*
    Clinicians generally perceived that delivering Dboxes to clinic nurses was useful. In one specific clinic, nurses had more responsibilities and were in charge of a significant portion of patient follow-up and phone calls, especially during more busy periods when physicians are less available (c52). In all settings, nurses mentioned often having to counsel patients on prenatal screening, and their intention to use the Dbox was in fact highest for this topic.
  4.9 *Government incentive*
    In one of the participating clinics, clinicians were receiving financial incentives from the government to screen patients for colorectal cancer. This was mentioned as a barrier to using the Dbox information with patients, mostly because such an incentive conveys the message that the authorities support colorectal cancer screening.

## Discussion

In this mixed methods implementation study of clinicians’ perceptions of eight Dboxes, which they received by email, clinicians had high intentions of using what they learned from the Dboxes to explain the pros and cons of the options to their patients. They felt that reading Dboxes improved their practice and that the information was relevant for at least one of their patients. Most were satisfied with the Dbox information, especially the figures and statistics that they valued as continuing professional development and for patient counselling. There was a consensus that to facilitate the presentation of options to their patients, the patients should get their own simplified decision aid, ideally before the consultation. The Dboxes that had been through prior testing were perceived as more valuable, suggesting that an iterative development could be required.

### Learning with the Dbox

Clinicians were generally satisfied with the Dboxes that offer specific questions to identify the decisional needs of patients and a bullet format to clearly present the information related to the available options, similarly to the symptom-based protocols they are used to, that provide specific questions to assess the severity of the problem and algorithms to identify the recommended level of health-care service [21].

Many clinicians did not make the leap from recognizing the equipoise to disclosing the need for a decision to their patients. The Dboxes may have brought to light certain undesirable aspects of their practice. The theory of cognitive consistency predicts that information which is compatible with existing beliefs is the most likely to be accepted, and that which emphasizes the undesirable qualities of existing beliefs may be selectively avoided [43]. This could also be because they thought their patients would have aversive reactions to dealing with uncertainty, a factor that is known to affect physicians’ willingness to engage in open communication about scientific uncertainty with their patients [44].

The strong emotions some participants expressed upon reading the Dboxes should not be interpreted as a negative effect of the intervention, as they could simply represent the first stage of an unfreeze-change-refreeze process. Unfreezing involves a disconfirmation of expectations and an induction of learning anxiety if the disconfirming data are accepted, an anxiety that can be converted into motivation to change [45].

### Sharing the Dbox information with patients

**Figure 2:**
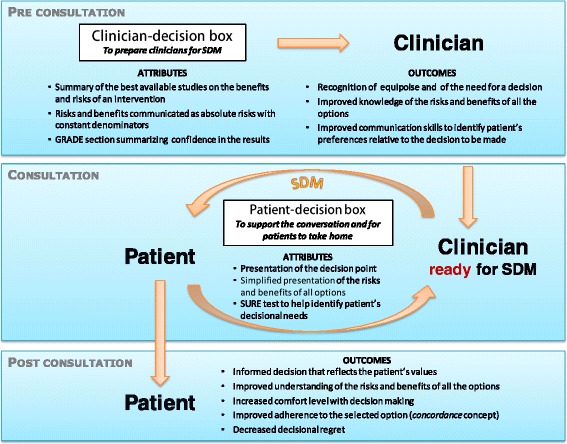
**Logic model of the tailored decision box approach combining clinician and patient versions of the decision box, with the mechanisms by which it supports shared decision-making (SDM).**

Clinicians emphasized that they would need a simplified tool to use in discussions with their patients or to hand out to read at home. Hence, based on the results of this project, we propose to tailor our approach by adding a simplified patient-decision box delivered to patients by their clinicians (Figure 2). This new component would fill a gap for rapid point-of-care clinical tools to facilitate clinician-patient communication [9,46]. From a theoretical perspective, the clinicians would then be the ones encouraging their patients to engage in a shared decision-making process, which concurs with a recent demonstration that a positive attitude of the clinician towards SDM increases patients’ willingness to engage in SDM [47].

Complex interventions combining patient and professional components appear to be a promising way to translate SDM into routine clinical practice. In a systematic review of 21 interventions to improve health professionals’ adoption of shared decision-making [5], only the three complex interventions comprising health professional training and a patient decision aid reported positive impacts [20,48,49]. Similarly, in a large study aiming to identify the factors differentiating effective and ineffective computerized clinical decision support systems, those systems providing advice to both practitioners and patients were more likely to be effective [50]. A few simple and brief tools already exist to support the provider-patient discussion at the point of care and could be used as co-interventions to the Dboxes [51-53].

Several of the barriers mentioned by participants were related to time constraints and patient preferences that were the most frequent external barriers reported in an earlier review of the barriers to implementing SDM in clinical practice [9].

### Dbox development process

The Prenatal and PSA Dboxes that had already been evaluated and optimized in a first study [25] were consistently better perceived by clinicians. This suggests that integrating users’ evaluation of each Dbox in a feedback loop is essential and that using the Dbox template alone, although helpful to optimize the overall design of the Dbox, is not sufficient to ensure that the content matches all of the user’s decision-making and reasoning skills and information-processing needs. Iterative development, one principle of usability testing, is used increasingly in health care [54-56] and allows users to partake in designing communication materials that are not only readable and understandable but also engaging and actionable [57].

### Implications of the results

In an earlier PtDA implementation study that used academic detailing to promote the distribution of PtDAs to patients, clinicians failed to distribute PtDAs because they did not believe that the decision was preference-sensitive [8]. There is thus a need for an intervention prior to asking clinicians to distribute PtDAs, to provoke a shift in their recognition of equipoise and of the need for a decision. To this end, Dboxes could be distributed to clinicians and staff playing an important role in the delivery of PtDAs [15]. This might limit the extent of the organizational commitment that is perceived as necessary in most studies of SDM implementation [10].

The Dbox template is valued by clinicians, so it is ready for use by producers of information resources, such as clinical practice guidelines, and systematic review developers to translate research information on the benefits and harms of health options to clinicians.

This project demonstrates the importance of end users’ involvement in the development of any communication interventions before implementation. Our work supports use of the IAM questionnaire to optimize evidence summaries as shown in previous research [58,59]. IAM was sensitive enough to detect differences in clinicians’ perceived value of the information, and its free-text comment box allowed us to better understand how to modify and improve Dboxes.

### Study limitations

As we do not have any information on the 40% of the invited clinicians who declined our invitation to participate in this study, we cannot ascertain whether participating clinicians were representative of the target audience. We also cannot extend our conclusions to specialized health care. To improve clinician participation in the next phase of this program, we plan to offer continuing professional development credits for each questionnaire completed, as credits represent a significant incentive for some physicians [26,27].

The participants felt that all possible topics should have been covered. With only eight Dboxes, they could not offer SDM consistent with all the decisions to be made by a single patient. It would be helpful if Dboxes covered all the available options as this would ensure they reach out to clinicians with different practice styles. For instance, clinicians who always use colonoscopy would not be as interested in a Dbox that compares screening with FOBT to no screening at all.

The measured questionnaire completion rate is relatively high compared to other similar studies [29]. However, we have not explored the reasons why participants did not look at the Dboxes in the first place, so we cannot rule out a selection bias if non-responders were less satisfied with the Dboxes than those who completed the questionnaires.

## Conclusions

Clinicians’ perceptions of the Dbox evidence summaries are promising, supporting the idea that we should start using this type of knowledge product to present research data for clinical questions where there is uncertainty that could lead to decisional conflict. However, to go one step further in the SDM process, we should evaluate whether the Dboxes really do support clinicians in involving their patients in SDM. We also need to test diverse development processes, such as using the IAM questionnaire for clinician feedback to optimize each tool or conducting individual interviews that are more resource intensive. We also need to confirm whether two iterations of user feedback are enough to optimize the Dboxes.

## Additional files

Additional file 1:
**Dbox sample.** The BRCA1/2 gene mutation test to evaluate the risks of breast and ovarian cancers. Additional file 2:
**Interview guides.** Structured interview guides used in **(a)** the focus groups with doctors, residents, and nurses, and **(b)** the individual interviews with the medical director of each clinic. Additional file 3:
**List of citations.** This file contains citations mentioned in the “Results” section. c1–c4 are clinic numbers.
