# Supplementary material for: Evidence summaries (decision boxes) to prepare clinicians for shared decision-making with patients: a mixed methods implementation study
Source: Implement Sci. 2014 Oct 5;9:144. doi: 10.1186/s13012-014-0144-6 (PMC4201673; doi:10.1186/s13012-014-0144-6)
Supplement: Additional file 1: — Dbox sample. The BRCA1/2 gene mutation test to evaluate the risks of breast and ovarian cancers. [file 13012_2014_144_MOESM1_ESM.pdf]

McMaster University | Université Laval | McGill

The BRCA1/2 gene mutation test to evaluate the risks of breast and ovarian cancer

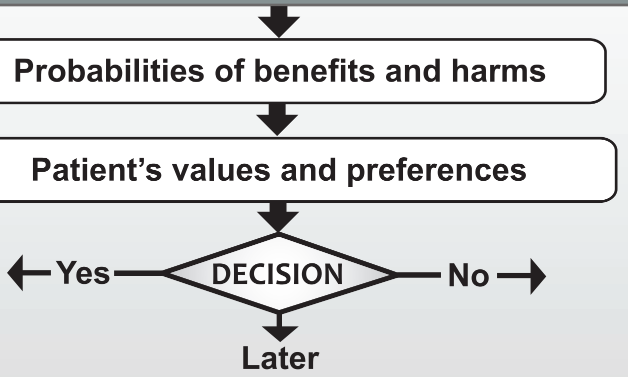

This document prepares the clinician to discuss scientific data with the patient so they can make an informed decision together.

Presenting the BRCA1/2 test to patients

What is this test for?

- This blood test **detects mutations of the BRCA1 and BRCA2 genes**. Individuals who carry these mutations have a **higher risk of developing breast and ovarian cancer.**

What options are available to mutation carriers?

1. Risk avoidance: increased physical activity and control of obesity, limiting alcohol consumption and dietary fat
2. Cancer screening or surveillance
3. Chemoprevention (e. g. tamoxifen, raloxifene)
4. Risk-reducing surgery (mastectomy, salpingo-oophorectomy)

Who might consider being tested?

1- Individuals who have a family history of:

- known BRCA1/2 mutation
- ovarian cancer in 2 or more relatives, at any age
- ovarian cancer at any age AND breast cancer under the age of 60 in 3 or more relatives
- breast cancer in 3 or more relatives average age of onset before age 50
- male breast cancer AND ovarian cancer[1]

2- Individuals of French-Canadian or Eastern European ancestry who are diagnosed with breast cancer under the age of 50, are diagnosed with ovarian cancer at any age, or have a first-degree relative who was diagnosed with breast or ovarian cancer under the age of 50.[1]

3- Individuals of Ashkenazi Jewish heritage diagnosed with breast cancer under the age of 65 or ovarian cancer at any age or with a first-degree relative affected by breast or ovarian cancer under the age of 65.[1]

- Individual risk for a BRCA1 or BRCA2 mutation can be calculated at https://www.myriadpro.com/brca-risk-calculator

Why do patient preferences matter when making this decision?

- **There are pros and cons to taking this test**

PROS: Individuals might **benefit from knowing that they carry a mutation** by taking steps to **reduce their cancer risk.**

CONS: At least **12%** of individuals who test negative for the mutation **may still develop breast cancer** and **1% may still develop ovarian cancer.**[2] From **35 to 90% of individuals with a BRCA1/2 gene mutation** (depending on the mutation and of type of cancer) **will not have breast or ovarian cancer,** so screening could lead to an **unnecessary invasive intervention that could have serious side effects** (see graphs, page 2).[3]

- **Both doing and not doing the test are acceptable options, so we propose that:**

1. the decision takes into account the **patient's values and preferences**
2. the clinician **shares this decision** with the patient

© Université Laval, 2012 all rights reserved

See page 2 for the current state of knowledge

DECISION BOX

BRCA1/2

State of knowledge – October 2011

Selection of best available studies

Benefits of screening

❶ Reassurance

For each 1000 individuals screened, **985 (98.5%)** will not have a mutation and can adhere to the general population screening guidelines. These individuals will be reassured.[4]

❷ Reduce cancer risks

For each 1000 individuals screened, **15 (1.5%)** will have a mutation detected. These individuals can choose to **take steps to reduce their risks** of having breast and/or ovarian cancer.[4]

Number of individuals with BRCA1/2 mutations found for each 1000 screened.[4]

Family history	General population	Personal history of cancer ↕	Ashkenazi Ancestry
without breast cancer < 50 years old or ovarian cancer in any relative	15	260	85
with breast cancer < 50 years old and/or ovarian cancer in at least a relative	70	575	230

↕ Breast cancer < 50 years old, and ovarian cancer at any age

Harms of screening

❶ False reassurance

At least 12% individuals **without** a BRCA1/2 mutation will develop breast cancer and **1%** will develop ovarian cancer at some point in their lifetime.[2] These individuals will have been **falsely reassured.**

- These proportions are greater in individuals with familial history of cancer or of BRCA1/2 mutation.

❷ False alarms (see graphs below)*

35-55% of BRCA1/2 mutation carriers will never develop **breast cancer** in their lifetime, and **60-90%** of BRCA1/2 mutation carriers will never develop **ovarian cancer**.[3]

❸ Overdiagnosis

Women identified as mutation carriers may use chemoprevention to reduce their risks. **10%** will undergo a **prophylactic bilateral mastectomy** and **40%** will undergo a **prophylactic salpingo-oophorectomy**.[5] These interventions reduce cancer risks but some treated women will still have cancer.

❹ Anxiety

Positive test results disclose genetic information about individuals and family members. This may create **anxiety** for individuals and **tension within families**.[6]

❺ Confidentiality

Individuals known to be mutation carriers may experience **discrimination** by employers or Insurance companies.[6]

☞ ***Confidence in the results:** **Moderate** The reported effect is inconsistent among trials. The study findings presented here are founded on a pooled analysis of 22 studies conducted in 12 countries with individuals carrying a broad spectrum of mutations.[3]

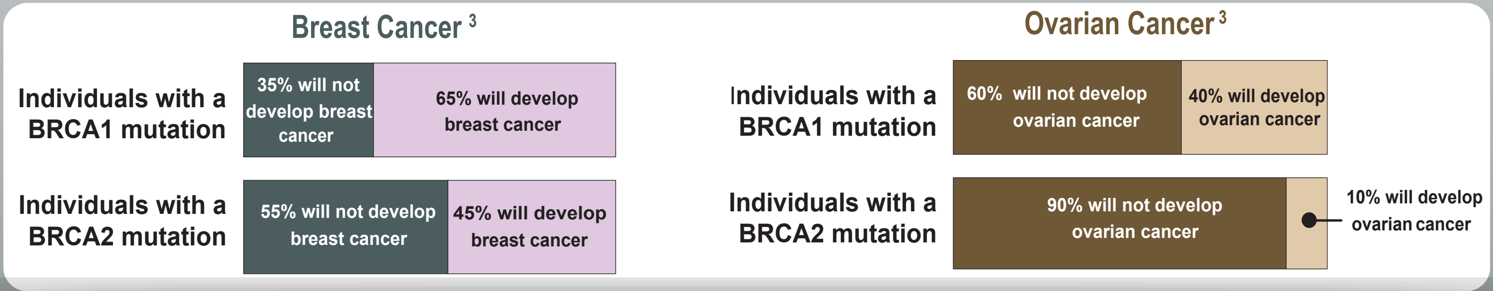

☞ Questions to identify the patient's decision making needs:

- Do you have any questions about the benefits and harms of each option?
- Which benefits and harms matter most to you?
- Do you feel sure about the best choice for you?
- Who will support and advise you in making a choice?

Study descriptions and references:

1. **McGill Cancer Genetics Program** 2006, http://www.mcgill.ca/files/cancergenetics/ReferralGuidelinesBreastOvarian.
2. **National Cancer Institute** 2010, http://seer.cancer.gov/faststats.
3. **Antoniou et al.** Am J Hum Genet 2003, 72(5), 1117-1130. **Study Design**: Systematic review of 22 case series studies in 12 countries. **Participants**: 8,000 individuals with breast/ovarian cancer (98% women, 2% men) unselected for family history. Of the 8000, 6% had a BRCA1/2 mutation. **Methods**: Cancer occurrence probabilities estimated with computer simulations.
4. **Myriad Genetic Laboratories Inc**. 2010. **Study Design**: Registry from a private molecular diagnostic company. **Participants**: 162,000 individuals from the U.S. who requested to be tested for the BRCA1/2 gene mutation.
5. **Domchek et al**. JAMA 2010, 304(9), 967-975. **Study Design**: Prospective multi-center cohort study assessing the relationship between risk-reducing mastectomy and risk-reducing salpingo-oophorectomy and cancer outcomes. **Participants**: 2,500 women with BRCA1/2 mutations from 22 clinical and genetics centres in North America and Europe. **Length of follow-up**: 4 years.
6. **Genetikit Research Team.** Gene Messenger 2010, http://www.cfp.ca/content/54/12/1691/suppl/DC1

© Université Laval, 2012 all rights reserved
