# Supplementary material for: Evidence summaries (decision boxes) to prepare clinicians for shared decision-making with patients: a mixed methods implementation study
Source: Implement Sci. 2014 Oct 5;9:144. doi: 10.1186/s13012-014-0144-6 (PMC4201673; doi:10.1186/s13012-014-0144-6)
Supplement: Additional file 2: — Interview guides. Structured interview guides used in (a) the focus groups with doctors, residents, and nurses, and (b) the individual interviews with the medical director of each clinic. [file 13012_2014_144_MOESM2_ESM.pdf]

Additional file 2

Structured interview guides used in (a) the focus groups with doctors, residents, and nurses, and (b) the individual interviews with the medical director of each clinic

(a) Focus groups with clinicians

- How did you use what you have learned from the decision boxes?
- How easy is it to use what you have learned from decision boxes in clinical encounters with patients?
- What resources are required for an appropriate application of what you have learned from the decision boxes?
- How can your clinic facilitate the use of what you have learned from the decision boxes in your everyday practice?
- Do you believe you can use the information presented in the decision boxes in a sustained manner (in the long run)?
- How does the way you work as a team influence your use of what you have learned from decision boxes in clinical encounters with patients?
- How does the information delivered by the decision boxes facilitate (or hinder) teamwork?
- According to you, what would be the best way to use the information delivered by decision boxes in primary care settings?
- Within your team, who do you think benefits the most from what you have learned from the decision boxes?

(b) Individual interviews with the clinics' medical directors

- How did the professionals from your team use what they have learned from the decision boxes?
- How easy do you think it is for the professionals from your team to use what they have learned from the decision boxes in clinical encounters with their patients?
- Do you believe that using the information presented in the decision boxes can be incorporated into your care practices in a sustained manner (in the long run)?
- How can your clinic facilitate use of the information provided in decision boxes in its practice? What internal resources at your clinic could be used to facilitate this use?
- What characteristics of your clinic and of the usual practice of your professionals, facilitate/hinder the use of what has been learned from the decision boxes?
- How is the daily workload of professionals affected by what has been learned from decision boxes in clinical encounters?
