# Supplementary material for: Evidence summaries (decision boxes) to prepare clinicians for shared decision-making with patients: a mixed methods implementation study
Source: Implement Sci. 2014 Oct 5;9:144. doi: 10.1186/s13012-014-0144-6 (PMC4201673; doi:10.1186/s13012-014-0144-6)
Supplement: Additional file 3: — List of citations. This file contains citations mentioned in the “Results” section. c1–c4 are clinic numbers. [file 13012_2014_144_MOESM3_ESM.pdf]

Additional file 3

List of citations mentioned in the Results section. Legend: C1-C4: clinic number

Citation number	Citation (source)
**c1**	"[...] Well, it's something that could be used for teaching. Or when we're reviewing cases with residents, it could sometimes come up. If, let's say, they have a patient who refuses the test that they think they should have, or vice versa, then that could be a way of getting the residents accustomed to this sort of thinking." (Focus group, C4)
**c2**	"What would be interesting for residents is that it could be evaluated for integration into critical reading activities." (Focus group, C2)
**c3**	"Seeing the boxes, one thing that attracted me was that I also felt like I was receiving a brief education [...] so if it they are presented not only as support for shared decisions, but also as a mechanism for continuing professional development, it might be easier [...]." (interview with clinic director, C1)
**c4**	"Great information, easy to read, to learn, and to teach. Patients love numbers to make decisions. Me too." (Web-questionnaire, ASA Dbox)
**c5**	"Nice addition to the information that I already give, particularly more accurate statistics when tests are positive or negative." (Web-questionnaire, Prenatal Dbox)
**c6**	"The statistics presented about the benefits are somewhat of a surprise to me, as they are relatively low....so this will help me in deciding how and who to encourage more or less to take medications given their personal circumstances, cardiovascular risks, etc." (Web-questionnaire, Statins Dbox)
**c7**	"[...] it's interesting how little benefits there are from a lot of the interventions that we all do all the time [...] And yet we keep on doing them over and over and over again." (Focus group, C3)
**c8**	"[...] I'm still kind of in a state of shock and digesting the information […]. It kind of puts me in a situation where I'm rethinking completely the notion of screening and what it means." (Focus group, C4)
**c9**	"[...] I had to stop looking at them because they just made me depressed. It seems like, you know, it doesn't matter which way you went, it wasn't going to do anything." (Focus group, C4)
**c10**	"Since there are few opportunities to reduce the progression of the disease, people need hope, and they often find little in the medication..." (Web-questionnaire, ChEIs)
**c11**	"Important to remember that the decision to treat does not rely only on numbers presented to a patient so he can decide. It is primarily a matter of clinical judgement (that no statistic can quantify) that takes into account, among other factors, age, degree of autonomy, ongoing medications, associated clinical conditions, etc. […]" (Web-questionnaire, Osteoarthritis Dbox).
**c12**	"[…] So I would use some of the... like some of the things in the PSA specifically), some of their targets in terms of how many would benefit versus how many would be harmed… the content, I would use the content with my discussion with the patient." (Focus group, C4)
**c13**	"The patient might say 'I've heard about colonoscopy so is this really a good test?' Then you could bring out the information and say 'well actually, it *is* a good test, and these are the numbers. This is how it's going to help you and these could be the harmful effects.'" (Director, C4)

Citation number	Citation (source)
**c14**	"[…] we were talking about her being on Lipitor. I think she was on 40 mg, and was wondering about a bit of muscle pain. So I said, 'we could lower your dose because your blood work is fine'. So we used the Dbox to kind of go through the pros and cons of it." (Focus group, C3)
**c15**	"I don't think I would use the actual pieces of paper or the tool stuff that's on the screen. But some of the information I would use with my discussions with patients to help come up with a plan." (Focus group, C4)
**c16**	"[…] I love the figures especially […]. I think it fits better because otherwise we are presenting generalities. I think that figures are more powerful and allow people perhaps to have a better idea." (Focus group, C1)
**c17**	"[…] which is where I think with the idea of the Dbox, you can present two very reasonable choices to the patient, of little consequence either way, in reality." (Focus group, C4)
**c18**	"We talked about it together. We chose it. It may not work. When we see each other again, there'll be no anger or aggression if it doesn't work. We clearly told them of the limits of medicine." (Focus group, C2)
**c19**	"Today, we share figures to try to see what is most important for the patients usually seen in family medicine. This is a step forward as people are much more accommodating with the decision. [...] In that context, we had 8 boxes that allowed us to have the information presented in a different or complementary way, to successfully explain to our patients certain concepts that are not always easy to understand [...]. This is why I find that it saves time and is easier. Compliance is improved." (interview with clinic director, C1)
**c20**	"It can affect compliance if the patient sees that it is useless..." (Web-questionnaire, Statins Dbox).
**c21**	"With this approach, it is hoped that the patient will be informed and will take a more satisfactory decision. We are wrong, in my opinion. We won't help him decide by showing him all the uncertainty." (Focus group, C2)
**c22**	"I tell you, in 22, 25 years of practice, I have never had a patient who asked me: 'How much? What percentage? What NNT do I have if I do the test or if I don't?' I present it to them like that, and patients will reflect using their own logic: it interests me or it doesn't interest me." (Focus group, C2)
**c23**	"I don't think I would use the actual pieces of paper or the tool stuff that's on the screen. But some of the information I would use in my discussions with patients to help come up with a plan." (Focus group, C4)
**c24**	"Document to help get us thinking, extremely interesting to share with patients and their spouses, and also enlightened us as professionals." (Web-questionnaire, Prenatal Dbox)
**c25**	"I would say that in my case, of the eight, there were three-quarters that I found difficult to use. I'm not sure I'd actually sit down with a patient to discuss the contents of it. [...] There are two, including screening for trisomy, which I found really straightforward, simple, synthetic, and easy to transmit." (Focus group, C2)
**c26**	"[…] What's interesting, or what's complicated about these, is that they're designed to be used in a context of shared decision making with the patient, but the Dboxes themselves are not written in a language that any patient can understand […]" (Focus group, C4)
**c27**	"[…] I would actually love if there was some very simplified version of this that the patient

Citation number	Citation (source)
	could read before they meet with you […]" (Focus group, C4)
**c28**	Speaker #1: "There's too much writing on the page. As I looked at it, I remember feeling for someone with a lower level of literacy or a lower level of education. The idea of quality of evidence is not very easy to understand, I think." Speaker #2: "Not at all. […]" (Focus group, C4)
**c29**	"[…] I was thinking—and I like your idea of proposing a simpler version—we could make it more visual in terms of less information and more pictures. Because I'm just thinking of how I'm going to try to speak to the patient. If I was to turn to them I'd want to show them what it looks like. I think it would be useful for them to have a visual representation of maybe what the risk might look like. Whether it's a pie chart or …" (Focus group, C4)
**c30**	"[…] the ideal would be to have two different Dbox formats, because we have at least two kinds of patients. We have patients who like statistics, who understand any explanation, data, risks, benefits, everything. But we also have a large percentage of patients who do not really understand, for whom it is really complex. Even for us, there are some very confusing issues. So for me, the ideal would be to have two different boxes. A more simplified and concise Dbox to explain to patients who have less understanding, and another one, like this one which is very well made. For me, it would be ideal." (Focus group, C1)
**c31**	"[…] I think it would be very interesting if the patient had an exercise to do before they came in, say, when you go to your doctor, we're going to make decisions together. The doctor may present some pros and cons and this is the model of care we're trying to perpetuate. Not like it used to be: the doctor up here, the patient down there, the doctor tells you what to do, you say yes. And you just do it. As opposed to now, where the approach to medicine is more a shared decision. Don't be surprised today if your doctor or nurse presents options to you and you have to come to a decision together […]" (Focus group, C4)
**c32**	"But if you really want to use these kinds of Dboxes, and you want to make it work, I think you could organize it so you would have a pre-visit." (interview with clinic director, C4)
**c33**	"I'm a little bit stuck on that one because when I'm reading it and I'm trying to digest the numbers that they're telling me, and the percentages, and how long you have to take it, and how many patients have to use it… it's a lot to digest." (interview with clinic director, C4)
**c34**	"[...] something that I can read overnight and when I return to my office, immediately, five minutes after, I can do something. With this, I am not able to do that." (Focus group, C2).
**c35**	"And it's a bit hard because a lot of the time I have this discussion with patients... and most of the time they're looking at me for my bottom line, like I'll say; 'it's up to you.' And then they come back to me; 'well, what do you think I should do?' So a lot of the time, patients aren't really... they want my guidance on it […]" (interview with clinic director, C4)
**c36**	"They don't care how many people need to be screened; they have it in their head that they want to be screened" (Focus group, C4)
**c37**	"I know all the family doctors talked about the 'time' thing, for sure, but I have tons of clients who are older patients so a lot of those things are hot topics, they come up all the time. But it seems like a huge thing to tackle to go through the stats." (Focus group, C3)
**c38**	Speaker #1: "I'm the opposite, I prefer having them online. I'm working a few places and I'd like to be able to access them anywhere [...]". Speaker #2: "He's that new wave guy who's like 'if it's not digital it's crap!'" (Focus group, C3)

Citation number	Citation (source)
**c39**	"[…] When we heard from the board of medicine: 'ear infections can be treated like this, sinusitis can be treated like that' ... for old doctors like me who were accustomed to giving the most commonly used drugs ... after a year, we followed their recommendations. It takes a year. In my case, it took me a year. Is it going to be the same for Dboxes? Maybe. We're not talking about a week, I can guarantee you that." (interview with clinic director, C2)
**c40**	"I think that's really the big thing: it's having a champion, and Dr. X is that champion for the Dbox. And I think that's probably the truth for most. Every time you try to institute some kind of practice change […], you do need someone who's going to be there, encouraging other people." (interview with clinic director, C4)
**c41**	"[…] Having journal clubs where everyone shares the same information, the same criticism, and even how to read certain information would facilitate using the information transmitted in the decision boxes. Some assessments of the quality of the act, concerning certain interventions, go against certain recommendations of your Dboxes. At some point, Dboxes would allow to be on the lookout for what needs to be discussed with our patients." (interview with clinic director, C2).
**c42**	"[...] for the new subjects, things I do less often, or with which I am less comfortable, I think it's a good starting point. And when there are any unclear things, it helps validate or verify [...]" (Focus group, C1)
**c43**	"[…] But for certain people, like you're following up on a bone density, you're in that grey area of whether to treat or not, that would be good. I would print it." (Focus group, C3)
**c44**	"[...] Because it really is the experience of patients that we need to know, their expectations and all that. When the figures take up too much space, I think we get lost [...]" (Focus group, C2)
**c45**	"[...] so it's a lot of topics that you're going to have to go through with the patient. And if in each one you have to sit and go through it and then you have to do the checkup, you're never going to get through it [...]. So there's a problem with the process, like in the sense that all of these topics are so relevant, you're never going to have time."(interview with clinic director, C4).
**c47**	"What I find is that for subjects with which I'm already comfortable or things I use a lot, I find it interesting, but I may use it a little less [...]" (Focus group, C1)
**c48**	Speaker #1: "As for trisomy 21, we have many opportunities to use the Dbox, but we already have a good tool with the screening program. I don't know if you've tried it..." Speaker # 2: "I would not change my ways." Speaker # 1: "The tool we have with the government screening program is already good, it is easy to explain." (Focus group, C1)
**c49**	"We work in a hospital environment, we're not the employers, we don't have enough support staff in. So you'd have to get support staff to say to the patient: 'Why are you coming in to see the doctor? … Oh, it's a checkup, then I'm going to book you with a nurse first, and then you'll see the doctor two weeks later'. I mean, we don't have appointments available two weeks later. There're so many barriers to this approach here, so it's not going to work. But if you really want to use these decision boxes, and you want to make it work, I think you could organize yourself that you would have a

Citation number	Citation (source)
	pre-visit." (interview with clinic director, C4)
**c50**	"Being a teaching unit, we train our residents in this way too. We’ve participated in several research projects so we’re accustomed to it: trisomy 21, informed decisions, antibiotics… so I think that, routinely, the medical profession is now this way." (interview with clinic director, C1)
**c51**	"[...] but we like to make decisions [...]. If we delay this, it will take another appointment, it will take time. But it is advisable to take the time to think." (Focus group, C1)
**c52**	"The nurses do so much in our clinic. They do a lot of visits with the patients. So when we're not available, they will see the patient. They will follow up if a patient has a particular problem or needs a followup. And if the physician is not available or it's appropriate for a nurse to do the followup, then they'll see the patient and follow up. They do phone calls; they do a lot of counselling... they do everything." (interview with clinic director, C4)
